# HPV-16 impairs the subcellular distribution and levels of expression of protein phosphatase 1γ in cervical malignancy

**DOI:** 10.1186/s12885-015-1141-0

**Published:** 2015-04-07

**Authors:** Takayuki Seiki, Kazunori Nagasaka, Christian Kranjec, Kei Kawana, Daichi Maeda, Hiroe Nakamura, Ayumi Taguchi, Yoko Matsumoto, Takahide Arimoto, Osamu Wada-Hiraike, Katsutoshi Oda, Shunsuke Nakagawa, Tetsu Yano, Masashi Fukayama, Lawrence Banks, Yutaka Osuga, Tomoyuki Fujii

**Affiliations:** 1 Department of Obstetrics and Gynecology, Faculty of Medicine, The University of Tokyo, Tokyo, 113-8655 Japan; 2 International Centre for Genetic Engineering and Biotechnology, Area Science Park, Padriciano-99, I-34012 Trieste, Italy; 3 Department of Pathology, Graduate School of Medicine, The University of Tokyo, Tokyo, 113-8655 Japan; 4 Department of Obstetrics and Gynecology, Graduate School of Medicine, Teikyo University, Tokyo, 173-8605 Japan; 5 Department of Obstetrics and Gynecology, National Center for Global Health and Medicine, Tokyo, 162-8655 Japan

**Keywords:** Cervical cancer, Immunohistochemistry, hScrib, Protein phosphatase 1, Proteasome degradation, Human papillomavirus 16

## Abstract

**Background:**

The high risk Human Papillomavirus (HPV) E6 oncoproteins play an essential role in the development of cervical malignancy. Important cellular targets of E6 include p53 and the PDZ domain containing substrates such as hScrib and Dlg. We recently showed that hScrib activity was mediated in part through recruitment of protein phosphatase 1γ (PP1γ).

**Methods:**

Expression patterns of hScrib and PP1γ were assessed by immunohistochemistry of HPV-16 positive cervical intraepithelial neoplasm (CIN), classified as CIN1 (n = 4), CIN2 (n = 8), CIN3 (n = 8), cervical carcinoma tissues (n = 11), and HPV-negative cervical tissues (n = 8), as well as by subfractionation assay of the HPV-16 positive cervical cancer cell lines, CaSki and SiHa. To explore the effects of the HPV-16 oncoproteins, we have performed siRNA knockdown of E6/E7 expression, and monitored the effects on the expression patterns of hScrib and PP1γ.

**Results:**

We show that PP1γ levels in HPV-16 positive tumour cells are reduced in an E6/E7 dependent manner. Residual PP1γ in these cells is found mostly in the cytoplasm as opposed to the nucleus where it is predominantly found in normal cells. We have found a striking concordance with redistribution in the pattern of expression (9/11; 81.8%) and loss of PP1γ expression in HPV-16 positive cervical tumours (2/11; 18.2%). Furthermore, this loss of PP1γ expression and redistribution in the pattern of expression occurs progressively as the lesions develop (8/8; 100%).

**Conclusion:**

Together, these results suggest that PP1γ may be a novel target of the HPV-16 oncoproteins and indicate that it might be a potential novel biomarker for HPV-16 induced malignancy.

## Background

Human Papillomaviruses (HPVs) are the aetiological agents of cervical cancer [1]. This is caused by infection with the high risk subset of HPV types, of which HPV-16 is the most important, being responsible for over 60% of global cervical cancer cases [2]. Cancer-causing HPVs encode two oncoproteins, E6 and E7, whose continued expression and activity is essential for maintaining the malignant phenotype, many years after the initial immortalising events [3,4]. Both viral oncoproteins function by perturbing the normal activity of a variety of different cellular control mechanisms. HPV E7 promotes cell cycle progression, in part through its association with members of the pocket protein family of tumour suppressors [5], whilst HPV E6 counteracts the pro-apoptotic effects of E7 through targeting the p53 tumour suppressor [6]. In both cases, the viral oncoproteins make efficient use of the cellular ubiquitin-proteasome machinery, with E7 targeting pRb through the cullin 2 ubiquitin ligase complex [7], whilst E6 uses the E6AP ubiquitin ligase to target p53 [8]. The effects of E6 and E7 are therefore cooperative, and this is reflected both in tissue culture systems, where they cooperate in the immortalisation of primary keratinocytes [9-11], and in animal models of tumourigenesis, where they cooperate in the induction of tumours in the skin and cervix [12,13].

Whilst targeting the pRb and p53 pathways is obviously very important for cervical tumourigenesis, it is also clear that E6 and E7 have a large number of other activities, many of which are also important for tumour development. In the case of high risk HPV E6 oncoproteins, an intriguing class of targets that appear to be important for HPV E6 induced malignancy are the PDZ (PSD/Dlg/ZO) domain containing substrates [14,15]. These are bound by E6 via a short stretch of amino acids within the extreme carboxy terminal region of the E6 oncoprotein. Most importantly, this PDZ binding motif (PBM) is only found in the high risk HPV E6 oncoproteins and is absent from the benign HPV E6 proteins [16,17]. Through this PBM, E6 can interact with a large number of cellular PDZ domain containing proteins, many of which are subject to E6-induced proteasomal degradation and E6-induced redistribution [16,18-21]. One of the most important of these targets is the cellular tumour suppressor hScrib. In Drosophila Scrib was originally identified as a potential tumour suppressor [22], and more recent studies in mammalian tissues also indicate tumour suppressive potential for hScrib. Loss of Scrib cooperates with c-Myc in the development of mammary carcinogenesis and Scrib also downregulates ERK signaling, with hScrib deregulation correlating with poor cancer prognosis [23-27]. In cervical tumourigenesis, hScrib patterns of expression are also perturbed as lesions develop, with hScrib being completely absent in many late stage tumours [28]. We recently found that hScrib could interact with PP1γ [29] a protein phosphatase that plays a critical role in controlling chromatin organization and also has an important role in the DNA damage response pathway [30,31] This suggested that PP1γ expression patterns in cervical tumourigenesis might likewise be perturbed. Therefore we initiated a series of studies to investigate the pattern of PP1γ expression in HPV16 positive cervical tumours and derived cell lines. We show that PP1γ is indeed subject to a striking alteration in both its levels of expression and localisation, both as lesions develop, and in the tumour derived cell lines. However this altered pattern of expression is independent of hScrib, is due directly to E6/E7 expression, and highlights PP1γ as potential novel biomarker of HPV induced neoplasia.

## Methods

### Cell lines and culture

HPV positive cervical cancer cell lines, CaSki, SiHa and HeLa plus HPV negative C33A (cervical cancer derived) and HaCaT (human keratinocytes) cells were cultured in Dulbecco’s modified Eagle’s medium (DMEM) supplemented with 10% fetal bovine serum at 37°C in a humidified incubator with 5% CO_2_ [32]. The effect of proteasome inhibitor was determined 24 hours post-transfection after 3 hours of treatment with 10 μM MG132 (Calbiochem).

For plasmid transfection, 293 cells were transfected using TransIT-293 transfection reagent (Mirus Bio) and HaCaT cells were transfected using Lipofectamine 2000 (Invitrogen), according to the manufacturer’s instructions, with pcDNA-HPV-16 E6. A plasmid expressing *β*-galactosidase was included in each transfection and pcDNA was used to equalize the input DNA.

### Antibodies

The following commercial antibodies were used at the dilution indicated: anti-hScrib goat polyclonal antibody (Santa Cruz WB 1:1000, IHC 1:100), anti-PP1γ goat polyclonal antibody (Santa Cruz WB 1:1000), anti-PP1 Gamma/PPP1CC Antibody LS-B4960 IHC-plus (tm) rabbit polyclonal antibody (Lifespan bioscience, Inc. IHC 1:200), anti-PP1γ sheep polyclonal antibody (Abcam, WB 1:1000), anti-actin monoclonal antibody (Sigma, WB 1:5000), mouse monoclonal anti-p53 (DO-1) (Santa Cruz WB 1:500), anti-p84 mouse monoclonal antibody (Abcam, WB 1:1000), anti-E-Cadherin rabbit polyclonal antibody (Santa Cruz WB 1:500), anti-α-tubulin mouse monoclonal antibody (Abcam, WB 1:1000), mouse monoclonal anti-vimentin antibody (Santa Cruz WB 1:500).

### siRNA transfection

The HPV-positive cervical cancer cells were seeded on 6 cm dishes and transfected using Lipofectamine 2000 (Invitrogen) with control siRNA against Luciferase (siLuc), or siRNA against HPV-16 and 18 E6 sequences (Dharmacon) described previously by Kranjec C et al., 2011. 72 hours post-transfection cells were harvested and total cell extracts or cell fractionated extracts were then analysed by western blotting. Alexa 568 labeled negative control siRNA (Qiagen) was used to measure transfection efficiency. The transfection efficiency was determined to be over 70% for each cell line.

### Subcellular fractionation assays

Differential extraction of the cells to obtain cytoplasmic, membrane, cytoskeleton, and nuclear fractions was performed using the Calbiochem Proteo Extract Fractionation Kit according to the manufacturer’s instructions. To inhibit phosphatase activity during the preparation of cell lysates, phosphatase inhibitors (1 mM Na_3_VO_4_, 1 mM β-Glycerophosphate, 2.5 mM Sodium Pyrophosphate, 1 mM Sodium Fluoride) were also included.

### Western blotting

Total cellular extracts were prepared by directly lysing cells from dishes in SDS lysis buffer. Alternatively cells were lysed in either E1A buffer (25 mM HEPES pH 7.0, 0.1% NP-40, 150 mM NaCl, plus protease inhibitor cocktail; Calbiochem) or RIPA buffer (50 mM Tris HCl pH 7.4, 1% NP-40, 150 mM NaCl, 1 mM EDTA, plus protease inhibitor cocktail; Calbiochem). For western blotting, 0.45 μm nitrocellulose membrane (Schleicher and Schuell) was used and membranes were blocked for 1 hour at 37°C in 10% milk/PBS followed by incubation with the appropriate primary antibody diluted in 10% milk/0.5% Tween 20 for 1 hour. After several washings with PBS 0.5% Tween 20, HRP-conjugated secondary antibodies (DAKO) in 10% milk/0.5% Tween 20 were incubated for 1 hour. Blots were developed using Amersham ECL reagents according to the manufacturer's instructions.

### Immunohistochemistry

All tissue samples were fixed in formalin and embedded in paraffin (obtained from patients under Institutional Review Board approval through the University of Tokyo Hospital). For all antibodies, immunostaining was performed according to standard techniques using an autostainer (BenchMark XT; Ventana Medical Systems, Inc., Tucson, AZ, USA). Immunoreactivity was interpreted based on the negative control, which was incubated without the primary antibody. Detection of hScrib expression was evaluated based on the existence of basolateral membrane staining as described previously [28]. For PP1γ, the expression was evaluated by nuclear staining. The immunostaining patterns of each sample were evaluated independently and blindly by pathologists specializing in gynaecological pathology, and cytology.

### PCR-based HPV DNA testing

DNA was extracted from cervical smear samples by using the QIAGEN® DNeasy® Blood & Tissue Kits. PCR-based HPV DNA testing was performed using the PGMY-CHUV assay. Briefly, standard PCR was conducted using the PGMY09/11 L1 consensus primer sets and HLA-dQ primer sets. Reverse blotting hybridization was subsequently performed as described previously [33].

## Results

### Distribution patterns of hScrib and PP1γ in HPV-16 positive cervical intraepithelial neoplasm (CIN) and cervical carcinoma tissues

**Figure 1:**
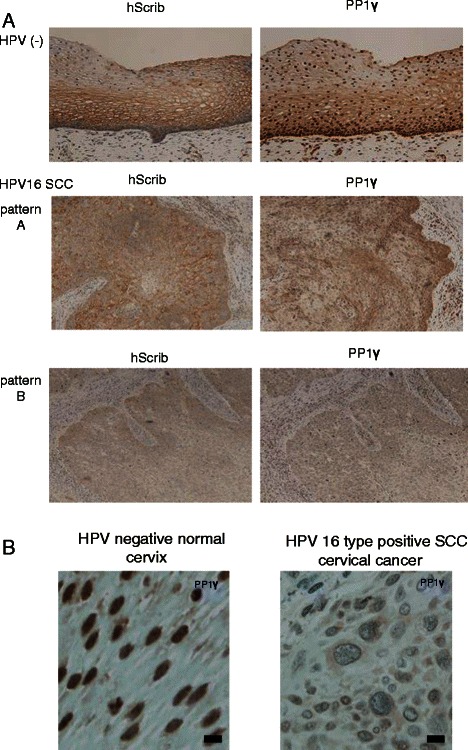
**Immunohistochemical analysis of the expression and localisation of hScrib and PP1γ in advanced squamous cervical carcinomas. (A)** Paraffin embedded excised tissues were immunostained with anti-hScrib or anti-PP1γ as indicated, and counterstained with haematoxylin. For the antibodies, immunostaining was performed according to standard techniques using an autostainer (BenchMark XT; Ventana Medical Systems, Inc., Tucson, AZ, USA). Representative experiments for a section of cervical epitheliums from normal cervix and advanced squamous cervical carcinomas (×200 original magnification). **(B)** High resolution microscopic images (scale bars: 20 μm) for a section of cervical epitheliums from normal cervix and HPV-16 positive advanced squamous cervical carcinomas.

Previous studies had highlighted hScrib as a potential biomarker for HPV-16 induced malignancy [19,28,34]. We reasoned that if PP1γ was also regulated directly by hScrib, this should be similarly affected in HPV-16 induced malignancy. In order to investigate this we performed IHC analysis of hScrib and PP1γ expression in HPV-16 positive cervical tumours and control cervix. The results obtained are shown in Figure 1 and Table 1. In normal tissue hScrib is found primarily at cell-cell junctions, with high levels of expression as the cells begin to differentiate. However, hScrib distribution is altered significantly in all the HPV-16 positive tumours, with significant redistribution in the pattern of expression in 5/11 tumours and a complete loss of expression in 6/11 tumours. These results are largely in agreement with previous studies [28,35]. In the case of PP1γ, this displays a largely nuclear pattern of expression and this is present throughout the differentiating epithelium in the normal cervical tissue. In contrast, in the cervical tumours there is a complete loss of expression of PP1γ in 2/11 cases, with a striking redistribution in the pattern of expression in the remaining 9 samples, where there was a shift from a nuclear localisation to a cytoplasmic pattern of expression.

**Table 1:** **Immunostaining patterns for hScrib and PP1γ in clinical samples of human uterine cervix**

hScrib		
	Normal	16-positive
Membrane	8	0
Cytoplasm	0	5
Nuclear	0	0
No expression	0	6
Total	8	11
**PP1y**		
	**HPV negative**	**16-positive**
Membrane	0	0
Cytoplasm	0	9
Nuclear	8	0
No expression	0	2
Total	8	11

**Figure 2:**
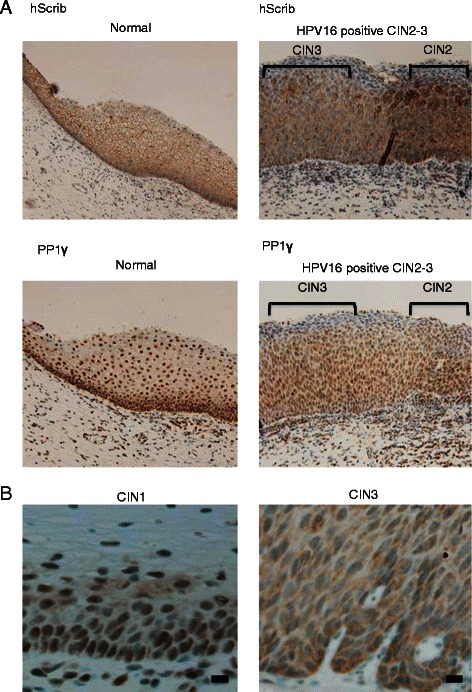
**Immunohistochemical analysis of the expression and localisation of hScrib and PP1γ in various stages of cervical intraepithelial neoplasms. (A)** Paraffin embedded excised tissues were immunostained with anti-hScrib or anti-PP1γ as indicated, and counterstained with haematoxylin. For the antibodies, immunostaining was performed according to standard techniques using an autostainer (BenchMark XT; Ventana Medical Systems, Inc., Tucson, AZ, USA). Representative experiments for a section of cervical epitheliums from normal cervix (left) and cervical intraepithelial neoplasms (CIN) grade 2 and 3 (right) (×200 original magnification). **(B)** High resolution microscopic images (scale bars: 20 μm) for a section of cervical epithelia from CIN grade 1 and 3.

We were then interested in investigating whether perturbation in the pattern of PP1γ expression was an early or late event during HPV-induced neoplastic progression. To do this we repeated the PP1γ IHC analysis on lesions exhibiting different grades of CIN. The lesions were classified as CIN1 (n = 4), CIN2 (n = 8), CIN3 (n = 8). As shown in Figure 2, there is a marked loss in nuclear PP1γ expression, which is already apparent in CIN2, and this is more evident in the CIN3 lesion, where there are also much lower levels of PP1γ expression. Interestingly, PP1γ positive cells were distributed only in the lower third of the epithelial layer in CIN1 cases (4/4) and 8/8 of patients with CIN3 had PP1γ positive cells distributed in the lower, middle, and upper third of the epithelium (Figure 2B). In the case of hScrib, there is a similar perturbation in the pattern of expression as the lesions develop, but similar to what has been reported previously, there is a tendency in some lower grade lesions to find highly overexpressed hScrib in regions of the epithelium.

These results indicate that hScrib and PP1γ, whilst both being perturbed during the progression to malignancy, are altered in a manner that is not interdependent, suggesting that PP1γ might be an independent marker for cervical tumour development. Indeed, the pattern and expression levels of PP1γ declined with an almost linear relationship from normal tissue, through increasing grades of CIN lesion, to invasive cancer.

### Analysis of PP1γ expression in HPV-16-positive cells

**Figure 3:**
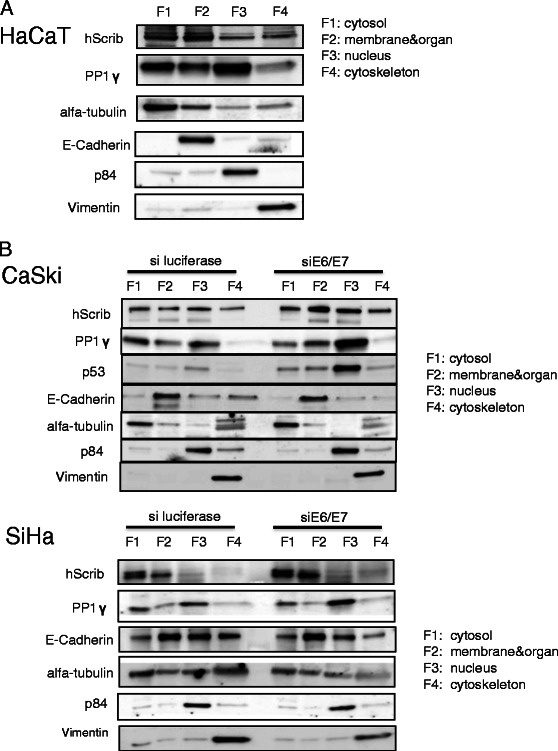
**PP1γ is mislocalised in HPV-16 positive tumour cells. (A)** HaCaT cells, and **(B)** siE6/E7or siluciferase control transfected CaSki and SiHa cells were fractioned into cytoplasmic (F1), membrane (F2), nuclear (F3), and cytoskeleton (F4) pools and hScrib and PP1γ were detected by western blotting. α-tubulin was a loading control for the cytoplasmic fraction, E-Cadherin was a loading control for the membrane fraction, p84 was a loading control for the nuclear fraction, and Vimentin was a loading control for the cytoskeleton fraction.

In order to determine whether perturbation of PP1γ expression was a direct result of HPV-16 oncoprotein function, we proceeded to examine the pattern of PP1γ expression in cell lines derived from HPV-16 positive cervical tumours. To do this we analysed the pattern of PP1γ expression in HPV-16 positive CaSki and SiHa cells, and compared this with HPV negative HaCaT cells. To determine whether any alterations might be HPV-specific, we also transfected the cells with siRNA E6/E7 and siLuc as a control. After 72 hours the cells were harvested and cells fractionated into cytosolic, membrane, nuclear and cytoskeletal pools, such that the pattern of PP1γ subcellular distribution could be monitored. The pattern of PP1γ expression was then ascertained by western blotting and the results obtained are shown in Figure 3. PP1γ is found predominantly within the nucleus in HaCaT cells (Figure 3A), whilst in the HPV-16 positive cells it is found weakly re-localised both in nuclear and cytoplasmic locations. However when E6/E7 expression is ablated there is a dramatic redistribution in the pattern of PP1γ expression, with much higher levels being found within the nuclear fraction of the cells (Figure 3B). In contrast, we found no difference in PP1γ transcript levels after siRNA E6/E7 treatment in HPV-16 positive cells (data not shown).

These results suggest that loss of nuclear PP1γ expression in HPV positive tumour cells is a direct result of the expression of the HPV E6/E7 oncoproteins.

### PP1γ is subject to degradation in HPV-16 positive cells

**Figure 4:**
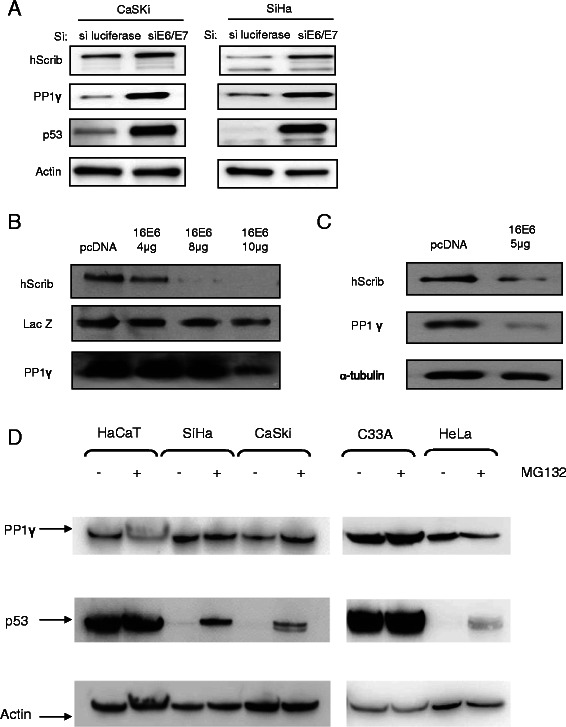
**PP1γlevels are downregulated in HPV16 positive cells by HPV E6/E7 oncogenes. (A)** HPV-16 positive CaSki and SiHa cells were transfected with siRNAE6/E7 or siLuc as control. Total cell extracts were then made after 72 hours, and hScrib, PP1γ, p53 and Actin were detected by western blotting. **(B)** 293 cells were transfected with 4, 8, 10 μg of HPV-16 E6 expression plasmid, and hScrib and PP1γ were analysed by Western blotting. The middle panel shows the LacZ transfection efficiency and loading control. **(C)** HaCaT cells were transfected with 5 μg of HPV-16 E6 expression plasmid, and hScrib and PP1γ were analysed by Western blotting. Tubulin was detected as control. **(D)** CaSki, SiHa, HeLa, C33A and HaCaT cells were incubated in the presence of either 10 μM MG132 or solvent before harvesting and analysed by western blotting. Actin was used as a loading control.

Interestingly, the fractionation studies indicate that whilst there is a significant increase in nuclear PP1γ in the absence of E6/E7, there is not a significant loss of cytoplasmic PP1γ, suggesting that some of the loss of nuclear expression may be due to proteasome mediated degradation. Therefore we were first interested in determining whether E6/E7 expression could affect the total levels of PP1γ expression. To do this we analysed the levels of PP1γ expression in total cell extracts from CaSki and SiHa cells previously transfected with siRNA E6/E7 or siLuc as a control. After 72 hours the cells were extracted and the levels of PP1γ expression monitored by western blotting. The results in Figure 4A show that loss of E6/E7 expression induces a marked increase in the total levels of PP1γ expression in HPV-16 positive cells, in a manner similar to that seen for restoration of p53 levels, which served as a positive control for efficient ablation of E6/E7 expression. We also monitored the efficiency of E6/E7 knockdown by RT-PCR and found that E6/E7 transcripts were reduced by around 60% following siRNA transfection (data not shown). In contrast to the change in PP1γ protein levels, we found no difference in PP1γ transcript levels after siRNA E6/E7 treatment in HPV-16 positive cells. Furthermore, to determine whether the cell type or E6 expression contributed to the alterations in PP1γ expression levels, we compared the ability of E6 to direct the degradation of PP1γ in 293 and HaCaT cells. First 293 cells were transfected with increasing amounts of HPV-16 E6, as indicated in Figure 4B. Then, we performed the same analysis using HaCaT cells (Figure 4C). The results demonstrated that overexpression of HPV-16 E6 results in a decrease in the level of PP1γ expression. In order to determine whether the loss of PP1γ expression was proteasome-mediated HPV-positive SiHa, CaSki and HeLa cells, and HPV-negative C33A and HaCaT cells were grown in the presence of the proteasome inhibitor, MG132 for 3 hours, after which the cells were harvested and the levels of PP1γ expression ascertained by western blotting. As can be seen from Figure 4D, there are minimal changes in the levels of PP1γ expression following proteasome inhibition, regardless of the presence or absence of HPV DNA sequences, whilst there is efficient rescue of p53 following proteasome inhibition in HPV positive cells. These results indicate that the effects of E6 upon PP1γ patterns of expression are most likely proteasome independent.

## Discussion

PP1 is a major serine/threonine protein phosphatase, normally regulating the phosphorylation status of a large number of important cellular regulatory proteins [36-39]. Important activities include the regulation of chromosome structure during mitosis and also following DNA damage, through de-phosphorylation of histones [30,40], and the control of centrosome disjunction through antagonism of Nek2A kinase activity [41].

In this study we have identified PP1γ as a potential new biomarker of HPV-16 induced malignancy. Using HPV-16 positive cervical tumour derived cell lines, IHC analysis of HPV-16 positive cervical tumours and CIN lesions, we present compelling evidence that PP1γ expression patterns are perturbed as a result of infection with HPV-16.

We originally considered that the PP1γ/hScrib complex might be a general target for HPV-16 E6, based on our previous studies showing complex formation between hScrib and PP1γ. However, analysis of the expression patterns of PP1γ and hScrib in cervical tissues indicate that this is not the case. Most importantly however, this highlights PP1γ as an independent target of the HPV-16 oncoproteins. In the normal cervix, PP1γ is expressed throughout the differentiating cervical epithelium, with a predominantly nuclear pattern of expression, which is consistent with previous studies [42]. To our surprise, we found that in all the HPV-16 positive cervical tumours analysed, this nuclear localisation of PP1γ was undetectable. Low levels of PP1γ can still be found within the cytoplasm of many cells within the majority of the cervical tumours that we analysed, although in 2/11 cases all PP1γ expression appeared to be lost. Similarly, perturbation in the pattern of PP1γ expression is apparent in CIN2 lesions, and this becomes more marked as the lesions progress to CIN3, suggesting that perturbation in the pattern of PP1γ expression is an early event in the development of cervical malignancy.

In order to understand whether these effects on PP1γ expression patterns were a direct consequence of E6/E7 activity, we then focused our attention on cells derived from HPV-16 positive cervical tumours. Again we found striking parallels with the IHC data, with very little PP1γ expression in the nucleus of HPV-16 positive CaSki or SiHa cells. In contrast, readily detectable nuclear PP1γ was observed in HaCaT cells. Most strikingly, siRNA ablation of E6/E7 expression resulted in a dramatic rescue of PP1γ expression within the nucleus of the HPV-16 positive cells, which appeared very similar to the effects seen upon the pattern of p53 expression. In contrast to p53 however, the alteration in the levels and pattern of PP1γ expression by E6 does not appear to involve the proteasome in cells derived from cervical tumours. Obviously further studies will be required to elucidate the precise mechanisms by which HPV-16 targets PP1γ.

## Conclusions

Currently we have no information as to whether the HPV-16 E6/E7 oncoproteins can modulate any of these phosphorylation events in a PP1γ dependent manner, it is nonetheless intriguing that all of these pathways are perturbed to some extent in cells containing the HPV-16 oncoproteins. Future studies will investigate these aspects further, but it is tempting to speculate that targeting of the nuclear forms of PP1γ might contribute directly towards the generation of genome instability, chromatin remodeling and tumour progression. The cellular redistribution of PP1γ seems to have an important role in the development of centrosome abnormalities and chromosomal instability at early stages of cervical carcinogenesis. Taken together this study highlights the potential value of PP1γ as a novel biomarker for HPV-induced cervical neoplasia.
